# A rare presentation of clinically diagnosed lyme disease with probable neuroborreliosis, septic shock, and bone marrow suppression: a case report

**DOI:** 10.1186/s12879-026-13899-y

**Published:** 2026-07-11

**Authors:** Yuyue Zhao, Hui Wang, Jun Duan

**Affiliations:** Department of Critical Care Medicine, China-Japan Friendship Hospital, No. 2 Yinghua East Street, Chaoyang District, Beijing, 100029 China

**Keywords:** Lyme disease, Septic shock, Neuroborreliosis, Lyme meningitis, Intracranial hypertension, Pure red cell aplasia, Thrombocytopenia, Bone marrow suppression, Intensive care unit

## Abstract

**Background:**

Lyme disease is rarely considered in critically ill patients from regions not routinely recognised as endemic. Severe presentations including septic shock, central nervous system involvement, and bone marrow suppression may therefore be difficult to recognise, particularly when laboratory confirmation is incomplete.

**Case presentation:**

A 60-year-old man from an inland province of northern China was admitted to the intensive care unit with four months of relapsing fever, acute delirium, respiratory distress, and septic shock. During admission, he developed recurrent high-grade fever with migratory erythematous rashes. Collateral history revealed a tick bite approximately 15 months earlier, followed by an expanding erythematous lesion compatible with erythema migrans. Cerebrospinal fluid showed markedly elevated opening pressure (>30 cmH2O), lymphocytic pleocytosis, elevated protein, and a normal CSF-to-serum glucose ratio, consistent with aseptic meningitis. Bone marrow aspiration showed pure red cell aplasia and megakaryocyte maturation arrest. Blood, urine, and cerebrospinal fluid cultures were negative, and metagenomic next-generation sequencing did not identify alternative pathogens. Lyme serology showed isolated IgM positivity with IgG negativity. Confirmatory two-tier testing and CSF Borrelia antibody testing were unavailable in our centre. This patient was therefore classified as clinically diagnosed Lyme disease with probable neuroborreliosis. Treatment with ceftriaxone and doxycycline was followed by defervescence and haematological recovery. At follow-up several weeks after discharge, the patient was afebrile, fully alert, and oriented.

**Conclusions:**

This case highlights an unusual severe presentation of clinically diagnosed Lyme disease with probable neuroborreliosis, intracranial hypertension, septic shock, and bone marrow suppression. Geographic origin should not preclude diagnostic consideration. In critically ill patients with unexplained fever, cytopenias, and dynamic cutaneous lesions, careful tick exposure history and bedside dermatological assessment may prove decisive where laboratory testing is inconclusive.

**Supplementary Information:**

The online version contains supplementary material available at https://doi.org/10.1186/s12879-026-13899-y.

## Background

Lyme disease (Lyme borreliosis) is a tick-borne zoonosis caused by Borrelia burgdorferi sensu lato complex spirochetes, primarily transmitted via Ixodes ticks. It is the most prevalent vector-borne illness in North America and Europe [1, 2]. In China, Lyme disease has emerged as a public health concern since the first confirmed human case in Heilongjiang Province in 1986, and has now been identified across 29 provinces, with the highest seroprevalence in forested northeastern and northwestern regions [3].

The clinical course of Lyme borreliosis is traditionally categorised into early localised, early disseminated, and late disseminated disease [4]. Early localised infection is marked by erythema migrans (EM). Without treatment, roughly 20% of patients progress to early disseminated disease with neurological, cardiac, or dermatological manifestations. Late disseminated disease typically manifests as mono- or oligoarticular arthritis, while neuroborreliosis occurs in approximately 10–15% of patients [5]. Among patients with neuroborreliosis, encephalitis is uncommon, estimated at around 3.3% in a Scandinavian cohort, and may leave residual neurological symptoms in more than half of affected patients [6]. Raised intracranial pressure can occur in Lyme meningitis and may affect visual function [7].

Haematological involvement in Lyme disease, including thrombocytopenia and anaemia, is recognised, but overt bone marrow suppression with pure red cell aplasia (PRCA) is rare [8]. Lyme disease presenting as septic shock requiring intensive care unit (ICU)-level care is uncommon and difficult to recognise. Borrelia spirochetes cannot be cultured by standard microbiological methods, and metagenomic next-generation sequencing (mNGS) may have limited sensitivity in late disseminated disease because of the low circulating spirochaetal burden [1, 2].

We report a case of clinically diagnosed Lyme disease with probable neuroborreliosis in a patient from an inland province of northern China, where Lyme disease is not routinely considered. The patient presented with migratory cutaneous lesions, lymphocytic meningitis with intracranial hypertension, bone marrow suppression, and septic shock. Because confirmatory two-tier serology and CSF/serum Borrelia antibody index testing were unavailable in our centre, the neurological diagnosis was classified as probable rather than laboratory-confirmed.

## Case Presentation

### Patient history and presenting complaint

A 60-year-old man from an inland province of northern China was admitted to the ICU in late 2025. He presented with intermittent fever lasting four months, which recurred and worsened approximately two weeks prior to admission. His past medical history included type 2 diabetes mellitus, hypertension, atherosclerosis, and urinary calculi. He reported no travel to regions typically considered endemic for Lyme disease.

About four months before ICU admission, he developed intermittent fever without an identifiable trigger and self-administered ibuprofen and oral cephalosporins. Approximately two months before ICU admission, during an earlier admission to a regional hospital, an elevated lactate dehydrogenase (LDH) level of 450 U/L was documented. He was diagnosed with systemic inflammatory response syndrome (SIRS) and started methylprednisolone (40 mg/day), with temporary fever control. When steroids were tapered to 8 mg/day shortly before ICU admission, high-grade fever recurred (Tmax 39.9 °C) with pharyngeal discomfort. He received methylprednisolone (40 mg/day) in the emergency department. Projectile vomiting, progressive dyspnoea, and delirium then prompted emergency ICU admission.

### Clinical findings on ICU admission

Vital signs indicated septic shock: temperature 37.5 °C, heart rate 115 bpm, respiratory rate 31 breaths/min, and blood pressure 151/52 mmHg requiring noradrenaline support (0.08 µg/kg/min). High-flow nasal oxygen was required for respiratory support; bilateral basal crackles were audible on auscultation. Neurological examination showed acute delirium, increased limb tone, nuchal rigidity, and an equivocal right-sided Babinski sign—findings indicating meningeal irritation and possible upper motor neuron involvement. No active cutaneous rash was present.

## Diagnostic investigations

### Laboratory findings

Full blood count showed leukocytosis (WBC 20.5 × 10⁹/L), anaemia (haemoglobin ~ 95 g/L), and severe thrombocytopenia (platelets decreasing from 125 to 20 × 10⁹/L). Inflammatory markers were markedly elevated: procalcitonin (PCT) > 20 ng/mL, C-reactive protein (CRP) 248.69 mg/L, ferritin 693.1 ng/mL, and interleukin-6 (IL-6) 622.1 pg/mL. Consumptive coagulopathy and elevated liver enzymes indicated multi-organ dysfunction. Blood and urine cultures were sterile. Comprehensive autoimmune, viral (Epstein–Barr virus, cytomegalovirus), and fungal serological panels were negative. Examination of the peripheral blood film showed no intra-erythrocytic parasites. Metagenomic next-generation sequencing (mNGS) of both peripheral blood and cerebrospinal fluid did not detect any bacterial, viral, or fungal pathogens, including the agents responsible for tick-borne co-infections such as *Anaplasma phagocytophilum*,* Ehrlichia spp.*,* Rickettsia spp.*,* and Babesia spp.*Dedicated tick-borne co-infection serology was not performed, and this remains a diagnostic limitation.

### Physical examination

**Fig. 1:**
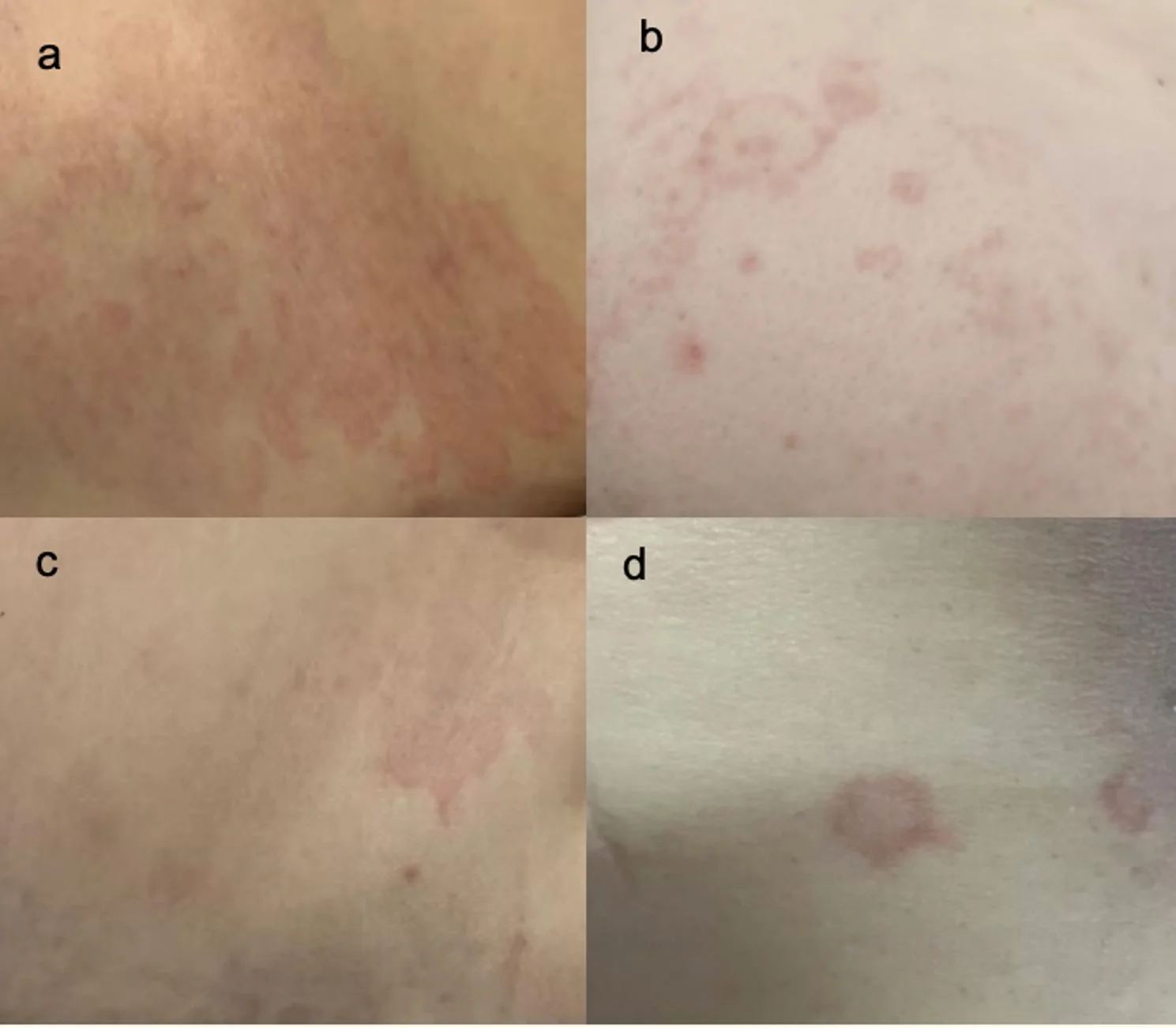
Clinical photographs taken during ICU admission showing the temporal evolution of cutaneous lesions in the reported patient. (**a**) Broad, confluent erythematous patches over the left lateral trunk. (**b**) Multiple discrete annular and serpiginous erythematous papules with surrounding faint macular erythema. (**c**) Partially resolved erythematous plaque with central clearing and a raised, expanding border. (**d**) Two well-demarcated annular erythematous lesions with prominent central clearing and an indurated ring. In the overall clinical context, these findings were considered compatible with erythema migrans-like lesions

Given the markedly elevated procalcitonin level and septic shock, methylprednisone therapy was discontinued in the ICU. Despite broad-spectrum antimicrobial therapy, the patient remained febrile. He developed sustained high-grade fever (39–40 °C), which responded poorly to antipyretics and physical cooling. During febrile episodes, diffuse erythematous rashes appeared over the trunk, neck, and proximal limbs. The lesions were non-pruritic, mildly raised, and mainly macular to annular with indistinct borders. They fluctuated with body temperature, becoming more prominent during fever and subsiding after defervescence (Fig. 1).

Collateral history from the family confirmed a tick bite to the periumbilical area during a rural outing approximately 15 months before intensive care admission. An expanding erythematous rash developed at the site several days later. The patient did not recognise the rash and did not seek medical advice at that time. Patients frequently overlook erythema migrans, especially on less visible body sites.The relapsing, migratory rash and the tick-bite history raised suspicion of a disseminated infectious process and prompted targeted serological testing for Borrelia burgdorferi.

Lyme serology was performed on serum separated from 3 mL of peripheral venous blood after centrifugation at 3,000 r/min for 10 min and storage at 4 °C before testing. Anti-Borrelia burgdorferi IgM and IgG antibodies were detected by enzyme-linked immunosorbent assay (ELISA) using a commercial kit from IBL International, Germany, according to the manufacturer’s instructions; the clinical laboratory reported isolated IgM positivity and IgG negativity. Given the 15-month interval from the erythema migrans-like rash, the isolated IgM-positive and IgG-negative ELISA result was interpreted as supportive but not confirmatory evidence [9].

Given the non-specific initial presentation, we first undertook a broad diagnostic work-up, including bacterial, viral, fungal, autoimmune, haematological, and mNGS investigations. After recurrent migratory erythematous lesions were observed in the ICU and collateral history of tick exposure with an erythema migrans-like rash was obtained, Lyme serology was requested. Guidelines emphasise that suspected Lyme disease should be assessed by integrating epidemiological exposure, compatible clinical manifestations, and specific serological evidence rather than by serology alone [1, 2]. In this case, the available evidence was a single qualitative ELISA result without confirmatory two-tier testing, so the result was interpreted as supportive evidence for clinically diagnosed Lyme disease rather than as stand-alone confirmation.

### Bone marrow aspiration

Positron emission tomography-computed tomography (PET-CT) demonstrated splenic and lymph node uptake that initially raised lymphoma as a differential diagnosis, which was subsequently excluded by bone marrow histopathology. Bone marrow aspiration showed an infectious and inflammatory marrow picture with PRCA and megakaryocyte maturation arrest. Reactive stromal hyperplasia was noted; overt haemophagocytosis was absent. Flow cytometry and immunophenotyping did not support lymphoma or leukaemia. Alternative causes of PRCA were systematically reviewed. ANA testing was negative. Parvovirus B19 serology and mNGS of both blood and CSF were negative.

### Neuroimaging and intracranial pressure assessment

Due to high fever and deteriorating consciousness, endotracheal intubation with subsequent mechanical ventilation was administered during the first week of ICU admission. Brain CT revealed hypodensities in the left occipital lobe and bilateral corona radiata, suggesting early ischaemic change; these findings alone were considered insufficient by the neurology team to explain the degree of impaired consciousness. Bedside optic nerve sheath diameter (ONSD) ultrasonography showed bilateral widening (left 0.738 cm, right 0.651 cm), and transcranial Doppler (TCD) revealed an elevated pulsatility index (PI > 1.2)--both indicative of raised intracranial pressure (ICP) [10]. These non-invasive ICP assessments supported the decision to proceed with lumbar puncture.

### Lumbar puncture

CSF opening pressure was markedly elevated at > 30 cmH_2_O. CSF analysis showed lymphocytic pleocytosis (95 cells/mm3; 90% mononuclear), elevated protein at 1.294 g/L, and a normal CSF-to-serum glucose ratio, consistent with aseptic lymphocytic meningitis [5]. CSF cultures, bacterial antigens, India ink staining, and mNGS were all negative, excluding tuberculous, fungal, and conventional bacterial meningitis. During this period, the patient developed refractory hypernatraemia, which did not respond adequately to conventional management. Given the coexistence of severe intracranial hypertension, continuous renal replacement therapy (CRRT) was initiated to allow gradual correction of serum sodium and controlled fluid removal. Following CRRT, sodium levels improved and neurological status partially stabilised.

### Differential diagnosis

Initial differential diagnoses included sepsis of unknown origin with a secondary bone marrow response; haematological malignancy, particularly lymphoma, in view of the persistent fever, splenomegaly, and increased uptake on PET-CT; autoimmune encephalitis; and tuberculous meningitis. These possibilities were assessed through flow cytometry, bone marrow biopsy, comprehensive autoimmune serological testing, microbiological cultures, cerebrospinal fluid studies, and mNGS.

Tick-borne co-infections were also considered. Anaplasma phagocytophilum was less likely because leucocytosis, rather than the expected leucopenia, was present. *Ehrlichia spp.* were not detected by blood or CSF mNGS. Babesiosis was not supported because the peripheral blood film showed no intra-erythrocytic parasites and the marrow picture was PRCA rather than haemolytic anaemia. Powassan virus infection was considered unlikely because the clinical pattern was dominated by migratory erythematous lesions, PRCA, and response to antibacterial therapy.These findings made probable Lyme borreliosis the most plausible working diagnosis, although other tick-borne infections and primary haematological disease could not be excluded with absolute certainty.

### Clinically diagnosed Lyme disease with probable Lyme neuroborreliosis

Serum Lyme enzyme immunoassay returned IgM-positive and IgG-negative. The working diagnosis was clinically diagnosed Lyme disease with probable neuroborreliosis, based on the retrospectively elicited tick exposure and erythema migrans-like rash, lymphocytic meningitis, neurological manifestations, bone marrow findings, exclusion of major alternatives, and clinical response to therapy. Septic shock with multi-organ dysfunction syndrome and secondary bone marrow failure comprising PRCA and thrombocytopenia were considered temporally associated manifestations, but causality cannot be proven from a single case.

**Fig. 2:**
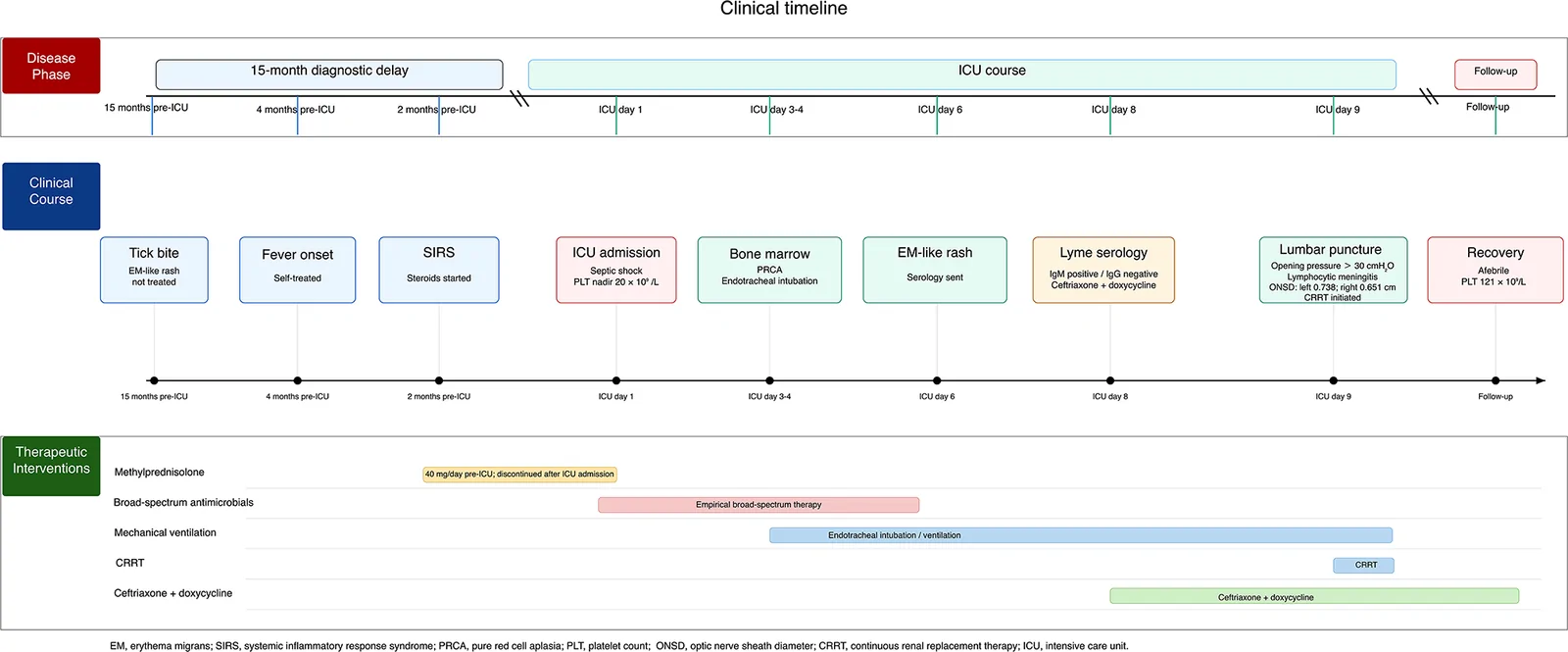
Clinical timeline illustrating the diagnostic delay and disease progression in a patient with clinically diagnosed Lyme disease with probable neuroborreliosis. The timeline depicts the course of illness from the initial tick bite through the prolonged diagnostic delay and ICU admission to eventual recovery. Calendar dates have been generalised to reduce patient identifiability. Abbreviations: EM, erythema migrans; SIRS, systemic inflammatory response syndrome; PRCA, pure red cell aplasia; PLT, platelet count; ONSD, optic nerve sheath diameter; CRRT, continuous renal replacement therapy; ICU, intensive care unit

The patient was treated with intravenous ceftriaxone (2 g once daily) and oral doxycycline (100 mg twice daily), with gradual defervescence and platelet recovery over the ensuing weeks. The clinical timeline illustrating the diagnostic delay and disease progression is shown in Fig. 2. At follow-up several weeks after discharge, the patient was afebrile, fully alert and oriented, and had achieved normalisation of platelet count.

## Discussion

### Epidemiological context: lyme disease in China

Since its initial documentation in 1986, Lyme disease in China has been confirmed across 29 provinces, with endemicity concentrated in forested northeastern provinces where Ixodes persulcatus is the principal vector [11]. A systematic review and meta-analysis of seropositivity data from 2005 to 2020 found an overall IgG seropositivity rate of 9.1% by enzyme immunoassay, with the highest burden in northeastern and western provinces [3]. Emerging evidence indicates an expanding geographic range for tick-borne diseases across inland Chinese provinces, driven by ecological and climatic shifts [11].

This patient came from an inland province not typically considered endemic for Lyme disease. His tick exposure occurred during a rural outing, which suggests that Lyme disease in China cannot be excluded on geographic grounds alone. The absence of a national Lyme disease surveillance programme in China, combined with limited clinician awareness outside traditionally endemic regions, contributed to the prolonged diagnostic delay in this case [3].

A key bedside finding was the recurrent, migratory erythematous rash during ICU admission. Unlike classic solitary erythema migrans in early localised infection, these lesions were multiple, transient, and dynamically distributed, with a clear temporal relationship to febrile episodes. The multidisciplinary team, including dermatology, infectious disease, and haematology, considered the pattern most compatible with secondary cutaneous manifestations of disseminated Lyme disease. Drug eruption, urticaria, and erythema multiforme were considered but were less consistent with the distribution, recurrence, fever association, and exposure history. Bedside observation of evolving skin signs, together with repeated history-taking, shifted the clinical assessment and prompted targeted testing.

### Clinically diagnosed Lyme disease presenting with septic shock

Septic shock attributable to *Borrelia burgdorferi* is rare and difficult to confirm. In this patient, IL-6 (622.1 pg/mL) and ferritin (693.1 ng/mL) indicated a severe inflammatory response. The response to ceftriaxone and doxycycline, together with the exclusion of alternative diagnoses, suggests *Borrelia burgdorferi* as a plausible central process. Because direct microbiological confirmation was unavailable, we describe septic shock as a major manifestation of clinically diagnosed Lyme disease rather than as definitively proven Borrelia sepsis.

### Haematological involvement: bone marrow suppression

Thrombocytopenia is the most frequently documented haematological abnormality in Lyme disease, arising through immune-mediated platelet destruction, direct spirochaetal effects on megakaryocyte progenitors, or inflammatory marrow suppression [8]. To our knowledge, the coexistence of Lyme disease and PRCA has not been previously reported. In this patient, bone marrow aspiration demonstrated erythroid hypoplasia with megakaryocyte maturation arrest and reactive stromal hyperplasia, without evidence of haemophagocytosis. Whether the bone marrow suppression was directly attributable to disseminated *Borrelia burgdorferi* infection or represented a concurrent process cannot be established from this single case.We present the bone marrow findings as a notable feature while acknowledging that the causal relationship with Lyme disease remains speculative.

### Probable neuroborreliosis with intracranial hypertension

Neuroborreliosis occurs in approximately 10–15% of Lyme disease patients and encompasses lymphocytic meningitis, cranial nerve palsies, painful radiculopathy, and encephalopathy [5]. Current guidelines recommend serum antibody testing in suspected Lyme neuroborreliosis and, when CSF is obtained for possible neurological involvement, paired CSF and serum testing using a validated CSF/serum antibody index to assess intrathecal Borrelia-specific antibody production [1, 2]. The CSF profile in this patient–lymphocytic pleocytosis with elevated protein and normal glucose–was compatible with aseptic meningitis and possible Lyme neuroborreliosis.

According to a Scandinavian systematic review and cohort study, encephalitis, defined by altered mental status, CSF pleocytosis, and intrathecal antibody production, was observed in only 3.3% of confirmed Lyme neuroborreliosis cases. This presentation was linked to longer diagnostic delays and a high frequency of residual symptoms (65.6% at one year). The authors suggest that atypical central nervous system involvement may go unrecognized far more often than previous reports would indicate [6]. Our patient’s encephalopathic features and prolonged diagnostic interval are compatible with this pattern, although intrathecal Borrelia antibody testing was not performed.

The markedly elevated opening pressure (> 30 cmH_2_O) represented a severe complication of lymphocytic meningitis. Bedside ONSD ultrasonography was used as a non-invasive ICP surrogate before lumbar puncture, a technique validated for detecting ICP > 20 mmHg (1 mmHg is approximately 1.36 cmH_2_O) [10]. The concurrent ischaemic changes on CT may be compatible with Lyme-associated cerebral vasculopathy, a recognised but infrequent complication of late neuroborreliosis resulting from inflammatory perivascular infiltration [5], but alternative critical-illness mechanisms cannot be excluded. In this patient, intracranial hypertension should therefore be considered possibly associated with probable Lyme neuroborreliosis.

The coexistence of hypernatraemia and intracranial hypertension posed a particular management challenge. In critically ill patients, hypernatraemia may result from dehydration, impaired central regulation, or treatment-related factors. Rapid correction carries a risk of cerebral oedema, especially in patients with neurological involvement. In this patient, CRRT provided a controlled method for gradual sodium correction and fluid management, helping to stabilise the patient’s condition.

### The serological paradox: IgM-positive, IgG-negative after 15 months

Standard Lyme disease serology uses a two-tier approach: initial enzyme immunoassay screening followed by confirmatory immunoblot or an equivalent second-tier assay [1, 2]. In immunocompetent individuals, IgM seroconversion usually occurs within 2–4 weeks, IgG seroconversion is expected by 4–6 weeks, IgM declines over subsequent months, and IgG may remain detectable for years despite successful treatment [9].

The atypical serological pattern must be interpreted in the immunological context of this case. The patient received prolonged corticosteroid exposure–methylprednisolone initiated during the pre-ICU illness and maintained for several weeks–during the period when IgG seroconversion would have been expected. Corticosteroids can suppress B-cell activation and proliferation, and high-dose methylprednisolone has been shown to reduce serum IgG concentrations through altered IgG synthesis and catabolism [12, 13]. This explanation remains inferential, but it provides a plausible context for delayed or blunted IgG generation. Taken together, the serology neither confirms nor excludes Lyme disease.

### Diagnostic challenges and lessons for clinical practice

Diagnosis was delayed because Lyme disease was not initially considered, the exposure history was obtained late, and early mNGS results were negative despite the limited sensitivity of this technique in late-stage Lyme borreliosis with low circulating spirochaetal burden. Prior corticosteroid exposure may also have suppressed the expected IgG response, making the serology harder to interpret.

In patients with unexplained fever, aseptic meningitis, and cytopenias, epidemiological history-taking should be included in the routine sepsis assessment. Particular attention should be paid to possible tick exposure and preceding skin lesions, even in areas where Lyme disease is not commonly recognised.

### Therapeutic approach

The 2020 IDSA/AAN/ACR guidelines recommend intravenous ceftriaxone (2 g once daily for 14 to 21 days) as the preferred regimen for neurological Lyme disease, with oral doxycycline as an effective alternative for selected neuroborreliosis presentations [1]. Given the severity of this case with septic shock, elevated ICP, and systemic hyperinflammation, we used a combination regimen of ceftriaxone and doxycycline. Although randomised evidence specifically supporting combination over monotherapy in severe Lyme disease is lacking, the pharmacokinetic rationale is complementary: ceftriaxone achieves therapeutic CSF concentrations with potent bactericidal activity against Borrelia, while doxycycline offers broad coverage against potential co-infecting tick-borne pathogens and carries additional anti-inflammatory properties. The clinical and haematological response was encouraging, but it should be interpreted as supportive rather than confirmatory evidence.

This case has several limitations. First, confirmatory two-tier Lyme serology, Western blot, and CSF/serum Borrelia antibody index testing were not available. We therefore classify the overall illness as clinically diagnosed Lyme disease with probable neuroborreliosis. Second, the serum Lyme test was a single qualitative ELISA reported by the clinical laboratory as IgM positive and IgG negative using an IBL International kit. Because the test was performed during routine clinical care, the numerical optical density/index value, antigen composition, lot-specific cut-off threshold, and confirmatory immunoblot result could not be retrieved retrospectively, limiting reproducibility. Third, specific serological testing for *Anaplasma phagocytophilum*,* Ehrlichia spp.*,* Rickettsia spp.*,* and Babesia spp.* was not performed because these test were not available in our centre. Although mNGS, peripheral blood film, and the clinical presentation did not support these co-infections, residual uncertainty remains.

## Conclusions

This case illustrates clinically diagnosed Lyme disease with probable neuroborreliosis, intracranial hypertension, septic shock, and bone marrow suppression. The diagnosis was supported by prior tick exposure, an erythema migrans-compatible lesion, recurrent migratory erythematous rashes, compatible CSF findings, exclusion of major alternatives, and improvement after Borrelia-targeted therapy. The isolated IgM-positive/IgG-negative serology and unavailable confirmatory testing limit laboratory certainty, but do not negate the clinical value of the exposure-rash history.

In critically ill patients with unexplained fever, cytopenias, aseptic meningitis, or dynamic skin lesions, careful exposure history and bedside dermatological assessment may be decisive, even where Lyme disease is not routinely suspected. Early targeted antimicrobial treatment may still be associated with favourable recovery in severe clinically supported cases.

## Supplementary Information

Below is the link to the electronic supplementary material.

Supplementary Material 1

## Data Availability

All data generated or analysed during this study are included in this published article and its supplementary information files.

## Declarations

### Use of AI writing tools

The authors used an AI-assisted writing tool (Claude, Anthropic) to assist with English language editing of a draft written entirely by the authors. All scientific content, clinical data, interpretation, and conclusions were generated solely by the authors. AI use is documented here in accordance with BMC policy; no AI tool has been listed as an author.

## Abbreviations

PRCA: Pure red cell aplasia
ICU: Intensive care unit
CSF: Cerebrospinal fluid
mNGS: Metagenomic next-generation sequencing
IgM: Immunoglobulin M
IgG: Immunoglobulin G
EM: Erythema migrans
CARE: CAse REport guidelines
LDH: Lactate dehydrogenase
SIRS: Systemic inflammatory response syndrome
WBC: White blood cell
PCT: Procalcitonin
CRP: C-reactive protein
IL-6: Interleukin-6
PET-CT: Positron emission tomography–computed tomography
ONSD: Optic nerve sheath diameter
TCD: Transcranial Doppler
PI: Pulsatility index
ICP: Intracranial pressure
PLT: Platelet count
CRRT: Continuous renal replacement therapy
HLH: Haemophagocytic lymphohistiocytosis
IDSA: Infectious Diseases Society of America
AAN: American Academy of Neurology
ACR: American College of Rheumatology
